# Supplementary material for: Fusion of crayfish optimization algorithm and MNS-YOLO for solar cell defect detection
Source: PLoS One. 2025 Oct 9;20(10):e0333939. doi: 10.1371/journal.pone.0333939 (PMC12510536; doi:10.1371/journal.pone.0333939)
Supplement: Attachment — Submitted filename: renamed_d0217.docx [file pone.0333939.s004.docx]

**Response to Reviewers**

**Fusion of crayfish optimization algorithm and MNS-YOLO for solar cell defect detection** (ID:PONE-D-25-02298R1)

Dear Editor Prof. Xu,

On behalf of all the co-authors, I would like to thank you for providing us with the opportunity to revise the manuscript. Following your comments, we have carefully revised the manuscript. Changes in the revised manuscript are marked in red. The responses to the reviewers’ comments are detailed below.

**Reviewer #1**:

The title of this article is "Fusion of crayfish optimization algorithm and MNS-YOLO for solar cell defect detection." It explores a new defect detection method aimed at improving the accuracy and efficiency of solar cell inspection.
Key Points:
1) Background: The article highlights the importance of defect detection in solar cells for enhancing photovoltaic system efficiency. Traditional methods have limitations, necessitating new algorithms to improve detection performance.
2) Methodology: The authors propose a method combining the crayfish optimization algorithm with the MNS-YOLO model, a deep learning-based object detection algorithm effective in identifying and locating various defects in solar cells.
3) Experimental Results: Tests on the PV-Multi-Defect dataset show that the CMNS-YOLO model outperforms other mainstream algorithms (e.g., YOLOv5, YOLOv6) in precision (P), recall (R), and mean average precision (mAP), demonstrating its effectiveness in solar cell defect detection.
4) Data Augmentation: To address dataset limitations, the article uses data augmentation techniques like flipping, rotation, contrast adjustment, and noise addition to enhance model robustness.
Comparison with EFS-YOLO:
EFS-YOLO has advantages in lightweight design, adaptability, resource efficiency, and experimental validation. It reduces model parameters and computational complexity through the EfficientViT backbone, CFE module, and shared parameter detection heads, improving real-time performance and edge device deployment. EFS-YOLO's robustness and versatility are validated on datasets like NEU-DET and GC10-DET, showing a good balance between lightweight design and detection accuracy. In contrast, CMNS-YOLO focuses more on network structure and target accuracy but lacks attention to lightweight design and versatility, limiting its application scope.

Comparison with YOLO-CXR:
"YOLO-CXR: A Novel Detection Network for Locating Multiple Small Lesions in Chest X-Ray Images" excels in several areas compared to the solar cell defect detection article. YOLO-CXR addresses the complex task of detecting multiple small lesions in chest X-rays, offering higher practical value for faster and more accurate diagnosis of chest diseases. It introduces innovations like RefConv layers, the Efficient Channel and Local Attention (ECLA) mechanism, and a dedicated small-lesion detection head, significantly improving feature extraction and small-lesion detection accuracy. Additionally, YOLO-CXR uses the large and diverse VinDr-CXR dataset, with rigorous experimental design and ablation studies, validating its effectiveness. In contrast, the solar cell defect detection article, while proposing improvements to MNS-YOLO, has fewer innovations, a smaller dataset, and simpler experimental design. Overall, YOLO-CXR demonstrates higher academic value and practical potential.
Drawbacks:
1) Long Training Time: The article notes that while the model shows significant advantages, training time is long for large datasets, potentially affecting practical application efficiency.
2) Limited Sample Size: Despite data augmentation, the original dataset has limited defect samples, which may impact the model's generalization ability.
3) Model Complexity: Combining multiple algorithms increases implementation complexity, requiring more computational resources and time in real-world applications.
4) Limitations: The article does not extensively discuss the model's performance under different environmental conditions. Future research could validate the model in more complex real-world scenarios.
In summary, this article provides a new approach to solar cell defect detection but faces challenges in practical application that need to be addressed.
References:
1. Chen B, Wei M, Liu J, et al. EFS-YOLO: a lightweight network based on steel strip surface defect detection[J]. Measurement Science and Technology, 2024, 35(11): 116003.
2. Hao S, Li X, Peng W, et al. YOLO-CXR: A novel detection network for locating multiple small lesions in chest X-ray images[J]. IEEE Access, 2024

**Response**: Thank you for your professional comments. We have taken your comments into consideration. First, we further validate the generalization ability of the model and the generality of the model on the public dataset of generic target detection, PVELAD (Please see P618, in red); second, we use the number of model parameters, GFLOPs, as a performance evaluation criterion for model lightweight, and use the number of frames per second, FPS, as an evaluation metric for the speed of the model detection in order to verify whether the model meets the requirements of real-time monitoring. (Please see P515,536,568, in red). Experiments are conducted to further verify that the model achieves high detection accuracy and generalization ability while maintaining a lightweight design.

We believe that these modifications, effectively highlight the academic value and practical potential of the CMNS-YOLO model proposed in this paper. We appreciate your suggestion as it helps to enhance the engineering value of this study.

**Reviewer #2**:

1. The dataset is relatively small; it is recommended to use a larger dataset in the future or conduct cross-dataset validation to further verify the model's generalization ability.

**Response**:Thank you for your professional comments. We have taken your comments into consideration and further validated the generalization ability of the model on the generalized target detection public dataset PVELAD. (Please see P618, in red).

These modifications, in our opinion, effectively highlight the significance of the CMNS-YOLO model proposed in this paper. We thank you for your suggestion as it helped us to further validate the validity as well as the generalization ability of the proposed model and to improve the clarity and persuasiveness of the results presented in the manuscript.

2.Compared with several mainstream object detection algorithms, CMNS-YOLO performs well in multiple indicators. However, it is not mentioned whether the hyperparameter settings of the baseline model have been optimized, and it is recommended to add details to ensure the fairness of the comparison experiment.

**Response**:Thank you for your specialized comments. We have considered your comments and combined the COA algorithm with YOLOv8n and MNS-YOLO, i.e., C-YOLO and CMNS-YOLO, respectively, and compared them with the baseline model YOLOv8n and MNS-YOLO networks to further verify the effectiveness of the COA algorithm in optimizing the network hyperparameters, and give the parameter changes before and after the model optimization. (Please see P584, in red).

We hope that this explanation will clarify the effectiveness of the COA algorithm in optimizing the hyperparameters of YOLO or modified YOLO and show the correlation between the values of the initial hyperparameters of the baseline model as well as the optimized hyperparameters and the performance metrics. We appreciate your suggestion as it helps to enhance the academic rigor of this study.

3.The images in the article are unclear, and furthermore, they should be inserted in the appropriate positions within the article.

**Response**:Thank you for your professional comments. We have taken your comments into consideration replaced the images in the original article with high-resolution images, in addition, and inserted them in the appropriate places in the article. By making these changes, we have improved the quality of the paper. We appreciate your suggestions as they help to improve the readability and quality of the manuscript.
