# Supplementary material for: Fusion of crayfish optimization algorithm and MNS-YOLO for solar cell defect detection
Source: PLoS One. 2025 Oct 9;20(10):e0333939. doi: 10.1371/journal.pone.0333939 (PMC12510536; doi:10.1371/journal.pone.0333939)
Supplement: Attachment — Submitted filename: resbose.docx [file pone.0333939.s005.docx]

**Response to Reviewers**

**Fusion of crayfish optimization algorithm and MNS-YOLO for solar cell defect detection** (ID: PONE-D-25-02298R1)

Dear Editor Prof. Xu,

On behalf of all the co-authors, I would like to thank you for providing us with the opportunity to revise the manuscript. Following your comments, we have carefully revised the manuscript. Changes in the revised manuscript are marked in red. The responses to the reviewers’ comments are detailed below.

**Editor:**

Thank you for your kind reminder regarding the journal’s requirements for the reference list. We have carefully reviewed all references to ensure their completeness and accuracy. Any necessary changes, including the replacement of outdated or non-English references with relevant current English-language sources, have been made and are clearly indicated in both the revised manuscript (highlighted in red) and in the rebuttal letter. We confirm that no retracted articles remain in the reference list without explicit justification, in full compliance with the journal’s publication standards.

**Journal Requirements:**

**Please review your reference list to ensure that it is complete and correct. If you have cited papers that have been retracted, please include the rationale for doing so in the manuscript text, or remove these references and replace them with relevant current references. Any changes to the reference list should be mentioned in the rebuttal letter that accompanies your revised manuscript. If you need to cite a retracted article, indicate the article’s retracted status in the References list and also include a citation and full reference for the retraction notice.**

**Response**:Thank you for your professional comments. We have carefully considered your suggestions. We have thoroughly reviewed the entire reference list to ensure its completeness and accuracy. In the process, we found that Reference 8 is an EI-indexed paper written only in Chinese, without an SCI version. Therefore, we have replaced it with the latest relevant English-language literature and revised the corresponding content in the manuscript accordingly (Please see P3, in red).

In addition, we have standardized and unified the formatting of all references to meet the requirements of PLOS ONE. The newly added references and the updated Reference 8 have been highlighted in red in the revised manuscript. We have ensured that the updated reference list is complete, accurate, and free of any retracted citations that have not been clearly indicated, fully complying with the journal’s publication standards.

We appreciate your attention and guidance.

**Reviewer #4**:

The manuscript proposes CMNS-YOLO, a model combining the crawfisher optimization algorithm with MNS-YOLO for solar cell defect detection. However, critical flaws in motivation, theoretical foundation, and experimental design undermine the validity of the research. Some suggestions are as follows:

Motivation: Recent YOLO variants [R1–R3] for surface defect detection are not explicitly compared and discussed. Moreover, the motivation solely relies on a literature review of solar cell defect detection without contextualizing the problem within broader challenges. It fails to link existing methods’ limitations. It would be better to extensively review the latest YOLO variants.

Technology: The paper omits background theorems for integrating the crawfisher algorithm with MNS-YOLO. It would be better to explain how the algorithm’s optimization strategy enhances YOLO’s feature extraction. Additionally, some key components lack clear roles when describing technology. Furthermore, the paper is not well organized, some equations sound quite messy.

Experiment: According to its response, the authors not well emphasize and address each comment raised by the reviewers. The generalization ability should be evaluated on some commonly used datasets such as NEU-DET and GC10-DET.
[R1] Usamentiaga, R., Lema, D. G., Pedrayes, O. D., & Garcia, D. F. (2022). Automated surface defect detection in metals: a comparative review of object detection and semantic segmentation using deep learning. IEEE Transactions on Industry Applications, 58(3), 4203-4213.
[R2] X. Huang, J. Zhu and Y. Huo, SSA-YOLO: An Improved YOLO for Hot-Rolled Strip Steel Surface Defect Detection, in IEEE Transactions on Instrumentation and Measurement, vol. 73, pp. 1-17, 2024, Art no. 5040017
[R3] Cui, L., Jiang, X., Xu, M., Li, W., Lv, P., & Zhou, B. (2021). SDDNet: A fast and accurate network for surface defect detection. IEEE Transactions on Instrumentation and Measurement, 70, 1-13.
[R4] Tian Y, Ye Q, Doermann D. Yolov12: Attention-centric real-time object detectors[J]. arXiv preprint arXiv:2502.12524, 2025.

**Response**:Thank you for your professional comments. We have carefully considered your suggestions.

First, regarding the motivation, we have expanded the main text to include a broader and more comprehensive review of the latest YOLO models and their variants, highlighting the limitations of existing approaches (Please see P7, in red). This situates the problem within a wider context of challenges, beyond relying solely on literature related to solar cell defect detection, and also takes into account the detailed design of the model. In future work, we will further clarify and comparatively discuss recent YOLO variants applied to surface defect detection.

Second, regarding the technical aspects, we have introduced and explained the rationale for integrating the Crayfish Optimization Algorithm with MNS-YOLO (Please see P23, in red). We have described in detail the core concept of using the Crayfish Optimization Algorithm to optimize MNS-YOLO’s hyperparameters, thereby reducing its sensitivity to parameter settings, preventing it from falling into local optima during training, and further enhancing its feature extraction capability (Please see P24, in red).

In addition, to more clearly describe the role of certain key components, we have rewritten these descriptions using more professional terminology and an improved academic writing style (Please see P14, P19, in red). Furthermore, to make the equations more professional and easier to understand, we have refined their formatting (Please see Equations 2, 5–9, 10, 12–16, 18, 20–24, in red) and updated the corresponding symbol representations.

Finally, with respect to the experiments, we plan to evaluate the generalization ability of our method on several commonly used datasets (such as NEU-DET and GC10-DET) in future work.

We believe these revisions strengthen the broader significance of the study, make the logical chain more complete, and enhance the technical soundness and credibility of our work. We appreciate your suggestions, which have helped us improve the quality of the manuscript and better convey its research value to readers in the field, providing a more meaningful reference for solar cell defect detection.
