# Supplementary material for: Fusion of crayfish optimization algorithm and MNS-YOLO for solar cell defect detection
Source: PLoS One. 2025 Oct 9;20(10):e0333939. doi: 10.1371/journal.pone.0333939 (PMC12510536; doi:10.1371/journal.pone.0333939)
Supplement: Attachment — Submitted filename: renamed_86c34.docx [file pone.0333939.s006.docx]

**Response to Reviewers**

**Fusion of crayfish optimization algorithm and MNS-YOLO for solar cell defect detection** (ID: PONE-D-25-02298R1)

Dear Editor Prof. Xu,

On behalf of all the co-authors, I would like to thank you for providing us with the opportunity to revise the manuscript. Following your comments, we have carefully revised the manuscript. Changes in the revised manuscript are marked in red. The responses to the reviewers’ comments are detailed below.

**Editor:**

Thank you for your kind reminder regarding the journal’s requirements for the reference list. We have carefully reviewed all references to ensure their completeness and accuracy. Any necessary changes, including the replacement of outdated or non-English references with relevant current English-language sources, have been made and are clearly indicated in both the revised manuscript (highlighted in red) and in the rebuttal letter. We confirm that no retracted articles remain in the reference list without explicit justification, in full compliance with the journal’s publication standards.

**Journal Requirements:**

**1. If the reviewer comments include a recommendation to cite specific previously published works, please review and evaluate these publications to determine whether they are relevant and should be cited. There is no requirement to cite these works unless the editor has indicated otherwise.
Response**: Thank you for your professional comment. We have considered your opinion. We have thoroughly and comprehensively reviewed and evaluated the reviewer's comments (including suggestions, references to previously published works), and determined that they are highly relevant to the content of this article.

**2. Please review your reference list to ensure that it is complete and correct. If you have cited papers that have been retracted, please include the rationale for doing so in the manuscript text, or remove these references and replace them with relevant current references. Any changes to the reference list should be mentioned in the rebuttal letter that accompanies your revised manuscript. If you need to cite a retracted article, indicate the article’s retracted status in the References list and also include a citation and full reference for the retraction notice.**

**Response**:Thank you for your professional comment. We have considered your opinion. We have thoroughly checked all reference lists to ensure their completeness and accuracy. In addition, we have checked and revised all the references raised by Reviewer 4 for any issues. In addition, all reference formats have been standardized and adjusted to comply with the requirements of PLOS ONE journal. The revised literature has been marked in red in the revised manuscript. We have ensured that the revised reference list is complete, accurate, and free of unexplained retracted references, meeting the publication requirements of the journal. Thank you for your attention and guidance.

**Reviewer #4**:

**Some equations should be formulated, such as Eqn. 25-28. As well, some terms are not well explained.**

**Regarding experimental environment: The experiments were conducted on PyTorch or Matlab?**

**It is recommended to add the explanation of A, B, C, D in the table captain (Table 3).**

**Most of references misuse "First Name" and "Last Name". Please check them all.**

**Response**:Thank you for your professional comment. We have considered your feedback and made the following modifications:

Firstly, we have checked all the formulas, carefully examined each parameter in the formulas, and added detailed terminology explanations for each parameter to improve the readability of the article and ensure that readers can accurately understand the meaning and function of each parameter.(Please see P17-P19) Secondly, regarding the experimental environment, we have revised the wording to clarify that all experiments were conducted using Python 3.8 and Pytorch 1.10.0 in Jupyter Notebook.(Please see P27) This modification clarifies the specific experimental environment, making it easier for readers to reproduce our research results. Then, we added explanations for A, B, C, and D in the table length of Table 3 to further help readers understand the different experimental groups, comparison conditions, and evaluation indicators represented by these labels, making the logic and content of Table 3 more complete.(Please see P32) In addition, we carefully examined the paper and made corresponding modifications to make its expression clearer and content more complete. (Please see P16,P30)Finally, we thoroughly examined and corrected the issue of improper use of names and surnames in the references to ensure that the citation format of author names conforms to academic standards, avoiding confusion caused by inconsistent naming, and enhancing the academic rigor of the reference section.(Please see P41-P52)

We would like to thank you again for your professional advice. We believe that through these revisions, not only has the quality of the paper been improved, but the accuracy of terminology expression has also been enhanced, and the research value can be clearly conveyed to readers in the field, providing a reference solution for the field of solar cell defect detection.
